# Supplementary material for: A Systematic Review of Educational Interventions for Informal Caregivers of People Living with Dementia in Low and Middle-Income Countries
Source: Behav Sci (Basel). 2024 Feb 24;14(3):177. doi: 10.3390/bs14030177 (PMC10968231; doi:10.3390/bs14030177)
Supplement: Supplementary file 1 [file behavsci-14-00177-s001.zip › behavsci-2839664-supplementary.pdf]

Supplementary Materials

Appendix - Search Terms

PsycINFO and MedLine (OVID)

1. exp Dementia/

2. (Alzheimer* or "Lewy body" or "Frontotemporal" or "Dement*" or "Vascular dementia" or "cognitive degeneration" or "cognitive impairment").mp. [mp=title, book title, abstract, original title, name of substance word, subject heading word, floating sub-heading word, keyword heading word, organism supplementary concept word, protocol supplementary concept word, rare disease supplementary concept word, unique identifier, synonyms]

3. (Carer* or Caregive*).mp. [mp=title, book title, abstract, original title, name of substance word, subject heading word, floating sub-heading word, keyword heading word, organism supplementary concept word, protocol supplementary concept word, rare disease supplementary concept word, unique identifier, synonyms]

4. (Famil* or informal or unpaid).mp. [mp=title, book title, abstract, original title, name of substance word, subject heading word, floating sub-heading word, keyword heading word, organism supplementary concept word, protocol supplementary concept word, rare disease supplementary concept word, unique identifier, synonyms]

5. (Support or Training or intervention or course or trial or group).mp. [mp=title, book title, abstract, original title, name of substance word, subject heading word, floating sub-heading word, keyword heading word, organism supplementary concept word, protocol supplementary concept word, rare disease supplementary concept word, unique identifier, synonyms]

6. (Awareness or Educa* or Psychoed*).mp. [mp=title, book title, abstract, original title, name of substance word, subject heading word, floating sub-heading word, keyword heading word, organism supplementary concept word, protocol supplementary concept word, rare disease supplementary concept word, unique identifier, synonyms]

7. (afghanistan OR albania OR algeria OR american samoa OR angola OR "antigua and barbuda" OR antigua OR barbuda OR argentina OR armenia OR armenian OR aruba OR azerbaijan OR bahrain OR bangladesh OR barbados OR republic of belarus OR belarus OR byelarus OR belorussia OR byelorussian OR belize OR british honduras OR benin OR dahomey OR bhutan OR bolivia OR "bosnia and herzegovina" OR bosnia OR herzegovina OR botswana OR bechuanaland OR brazil OR brasil OR bulgaria OR burkina faso OR burkina fasso OR upper volta OR burundi OR urundi OR cabo verde OR cape verde OR cambodia OR kampuchea OR khmer republic OR cameroon OR cameron OR cameroun OR central african republic OR ubangi shari OR chad OR chile OR china OR colombia OR comoros OR comoro islands OR iles comores OR mayotte OR democratic republic of the congo OR democratic republic congo OR congo OR zaire OR costa rica OR "cote d’ivoire" OR "cote d’ ivoire" OR cote divoire OR cote d ivoire OR ivory coast OR croatia OR cuba OR cyprus OR czech republic OR czechoslovakia OR djibouti OR french somaliland OR dominica OR dominican republic OR ecuador OR egypt OR united arab republic OR el salvador OR equatorial guinea OR spanish guinea OR eritrea OR estonia OR eswatini OR swaziland OR ethiopia OR fiji OR gabon OR gabonese republic OR gambia OR "georgia (republic)" OR georgian OR ghana OR gold coast OR gibraltar OR greece OR grenada OR guam OR guatemala OR guinea OR guinea bissau OR guyana OR british guiana OR haiti

OR hispaniola OR honduras OR hungary OR india OR indonesia OR timor OR iran OR iraq OR isle of man OR jamaica OR jordan OR kazakhstan OR kazakh OR kenya OR "democratic people's republic of korea" OR republic of korea OR north korea OR south korea OR korea OR kosovo OR kyrgyzstan OR kirghizia OR kirgizstan OR kyrgyz republic OR kirghiz OR laos OR lao pdr OR "lao people's democratic republic" OR latvia OR lebanon OR lebanese republic OR lesotho OR basutoland OR liberia OR libya OR libyan arab jamahiriya OR lithuania OR macau OR macao OR republic of north macedonia OR macedonia OR madagascar OR malagasy republic OR malawi OR nyasaland OR malaysia OR malay federation OR malaya federation OR maldives OR indian ocean islands OR indian ocean OR mali OR malta OR micronesia OR federated states of micronesia OR kiribati OR marshall islands OR nauru OR northern mariana islands OR palau OR tuvalu OR mauritania OR mauritius OR mexico OR moldova OR moldovian OR mongolia OR montenegro OR morocco OR ifni OR mozambique OR portuguese east africa OR myanmar OR burma OR namibia OR nepal OR netherlands antilles OR nicaragua OR niger OR nigeria OR oman OR muscat OR pakistan OR panama OR papua new guinea OR new guinea OR paraguay OR peru OR philippines OR philipines OR phillipines OR phillippines OR poland OR "polish people's republic" OR portugal OR portuguese republic OR puerto rico OR romania OR russia OR russian federation OR ussr OR soviet union OR union of soviet socialist republics OR rwanda OR ruanda OR samoa OR pacific islands OR polynesia OR samoan islands OR navigator island OR navigator islands OR "sao tome and principe" OR saudi arabia OR senegal OR serbia OR seychelles OR sierra leone OR slovakia OR slovak republic OR slovenia OR melanesia OR solomon island OR solomon islands OR norfolk island OR norfolk islands OR somalia OR south africa OR south sudan OR sri lanka OR ceylon OR "saint kitts and nevis" OR "st. kitts and nevis" OR saint lucia OR "st. lucia" OR "saint vincent and the grenadines" OR saint vincent OR "st. vincent" OR grenadines OR sudan OR suriname OR surinam OR dutch guiana OR netherlands guiana OR syria OR syrian arab republic OR tajikistan OR tadjikistan OR tadzhikistan OR tadzhik OR tanzania OR tanganyika OR thailand OR siam OR timor leste OR east timor OR togo OR togolese republic OR tonga OR "trinidad and tobago" OR trinidad OR tobago OR tunisia OR turkey OR turkmenistan OR turkmen OR uganda OR ukraine OR uruguay OR uzbekistan OR uzbek OR vanuatu OR new hebrides OR venezuela OR vietnam OR viet nam OR middle east OR west bank OR gaza OR palestine OR yemen OR yugoslavia OR zambia OR zimbabwe OR northern rhodesia OR global south OR africa south of the sahara OR sub-saharan africa OR subsaharan africa OR africa, central OR central africa OR africa, northern OR north africa OR northern africa OR magreb OR maghrib OR sahara OR africa, southern OR southern africa OR africa, eastern OR east africa OR eastern africa OR africa, western OR west africa OR western africa OR west indies OR indian ocean islands OR caribbean OR central america OR latin america OR "south and central america" OR south america OR asia, central OR central asia OR asia, northern OR north asia OR northern asia OR asia, southeastern OR southeastern asia OR south eastern asia OR southeast asia OR south east asia OR asia, western OR western asia OR europe, eastern OR east europe OR eastern europe OR developing country OR developing countries OR developing nation? OR developing population? OR developing world OR less developed countr* OR less developed nation? OR less developed population? OR less developed world OR lesser developed countr* OR lesser developed nation? OR lesser developed population? OR lesser developed world OR under developed countr* OR under developed nation? OR under developed population? OR under developed world OR underdeveloped countr* OR underdeveloped nation? OR underdeveloped population? OR underdeveloped world OR middle income countr* OR middle income nation? OR middle income population? OR low income countr* OR low income nation? OR low income population? OR lower income countr* OR lower income nation? OR lower income population? OR underserved countr* OR underserved

nation? OR underserved population? OR underserved world OR under served countr* OR under served nation? OR under served population? OR under served world OR deprived countr* OR deprived nation? OR deprived population? OR deprived world OR poor countr* OR poor nation? OR poor population? OR poor world OR poorer countr* OR poorer nation? OR poorer population? OR poorer world OR developing econom* OR less developed econom* OR lesser developed econom* OR under developed econom* OR underdeveloped econom* OR middle income econom* OR low income econom* OR lower income econom* OR low gdp OR low gnp OR low gross domestic OR low gross national OR lower gdp OR lower gnp OR lower gross domestic OR lower gross national OR lmic OR lmics OR third world OR lami countr* OR transitional countr* OR emerging economies OR emerging nation?).ti,ab,sh,kf.

8. 1 and 2 and 3 and 4 and 5 and 6 and 7

Web of Sciences

Dementia OR Alzheimer* OR “Lewy bod*” OR “Frontotemporal” OR “Dement*” OR “Vascular dementia” OR “cognitive* degeneration” OR “cognitive impairment” (Topic) and Carer* OR Caregive* (Topic) and Support or Training or intervention or course or trial or education or awareness (Topic) and afghanistan or albania or algeria or american samoa or angola or "antigua and barbuda" or antigua or barbuda or argentina or armenia or armenian or aruba or azerbaijan or bahrain or bangladesh or barbados or republic of belarus or belarus or byelarus or belorussia or byelorussian or belize or british honduras or benin or dahomey or bhutan or bolivia or "bosnia and herzegovina" or bosnia or herzegovina or botswana or bechuanaland or brazil or brasil or bulgaria or burkina faso or burkina fasso or upper volta or burundi or urundi or cabo verde or cape verde or cambodia or kampuchea or khmer republic or cameroon or cameron or cameroun or central african republic or ubangi shari or chad or chile or china or colombia or comoros or comoro islands or iles comores or mayotte or democratic republic of the congo or democratic republic congo or congo or zaire or costa rica or "cote d’ivoire" or "cote d’ ivoire" or cote divoire or cote d ivoire or ivory coast or croatia or cuba or cyprus or czech republic or czechoslovakia or djibouti or french somaliland or dominica or dominican republic or ecuador or egypt or united arab republic or el salvador or equatorial guinea or spanish guinea or eritrea or estonia or eswatini or swaziland or ethiopia or fiji or gabon or gabonese republic or gambia or "georgia (republic)" or georgian or ghana or gold coast or gibraltar or greece or grenada or guam or guatemala or guinea or guinea bissau or guyana or british guiana or haiti or hispaniola or honduras or hungary or india or indonesia or timor or iran or iraq or isle of man or jamaica or jordan or kazakhstan or kazakh or kenya or "democratic people’s republic of korea" or republic of korea or north korea or south korea or korea or kosovo or kyrgyzstan or kirghizia or kirgizstan or kyrgyz republic or kirghiz or laos or lao pdr or "lao people's democratic republic" or latvia or lebanon or lebanese republic or lesotho or basutoland or liberia or libya or libyan arab jamahiriya or lithuania or macau or macao or republic of north macedonia or macedonia or madagascar or malagasy republic or malawi or nyasaland or malaysia or malay federation or malaya federation or maldives or indian ocean islands or indian ocean or mali or malta or micronesia or federated states of micronesia or kiribati or marshall islands or nauru or northern mariana islands or palau or tuvalu or mauritania or mauritius or mexico or moldova or moldovian or mongolia or montenegro or morocco or ifni or mozambique or portuguese east africa or myanmar or burma or namibia or nepal or netherlands antilles or nicaragua or niger or nigeria or oman or muscat or pakistan or panama or papua new guinea or new guinea or paraguay or peru or philippines or philipines or phillipines or phillippines or poland or "polish people's republic" or portugal or portuguese republic or puerto rico or romania or russia or russian federation or ussr or soviet union or union of soviet socialist republics or rwanda or

ruanda or samoa or pacific islands or polynesia or samoan islands or navigator island or navigator islands or "sao tome and principe" or saudi arabia or senegal or serbia or seychelles or sierra leone or slovakia or slovak republic or slovenia or melanesia or solomon island or solomon islands or norfolk island or norfolk islands or somalia or south africa or south sudan or sri lanka or ceylon or "saint kitts and nevis" or "st. kitts and nevis" or saint lucia or "st. lucia" or "saint vincent and the grenadines" or saint vincent or "st. vincent" or grenadines or sudan or suriname or surinam or dutch guiana or netherlands guiana or syria or syrian arab republic or tajikistan or tadjikistan or tadzhikistan or tadzhik or tanzania or tanganyika or thailand or siam or timor leste or east timor or togo or togolese republic or tonga or "trinidad and tobago" or trinidad or tobago or tunisia or turkey or turkmenistan or turkmen or uganda or ukraine or uruguay or uzbekistan or uzbek or vanuatu or new hebrides or venezuela or vietnam or viet nam or middle east or west bank or gaza or palestine or yemen or yugoslavia or zambia or zimbabwe or northern rhodesia or global south or africa south of the sahara or sub-saharan africa or subsaharan africa or africa, central or central africa or africa, northern or north africa or northern africa or magreb or maghrib or sahara or africa, southern or southern africa or africa, eastern or east africa or eastern africa or africa, western or west africa or western africa or west indies or indian ocean islands or caribbean or central america or latin america or "south and central america" or south america or asia, central or central asia or asia, northern or north asia or northern asia or asia, southeastern or southeastern asia or south eastern asia or southeast asia or south east asia or asia, western or western asia or europe, eastern or east europe or eastern europe or developing country or developing countries or developing nation? or developing population? or developing world or less developed countr* or less developed nation? or less developed population? or less developed world or lesser developed countr* or lesser developed nation? or lesser developed population? or lesser developed world or under developed countr* or under developed nation? or under developed population? or under developed world or underdeveloped countr* or underdeveloped nation? or underdeveloped population? or underdeveloped world or middle income countr* or middle income nation? or middle income population? or low income countr* or low income nation? or low income population? or lower income countr* or lower income nation? or lower income population? or underserved countr* or underserved nation? or underserved population? or underserved world or under served countr* or under served nation? or under served population? or under served world or deprived countr* or deprived nation? or deprived population? or deprived world or poor countr* or poor nation? or poor population? or poor world or poorer countr* or poorer nation? or poorer population? or poorer world or developing econom* or less developed econom* or lesser developed econom* or under developed econom* or underdeveloped econom* or middle income econom* or low income econom* or lower income econom* or low gdp or low gnp or low gross domestic or low gross national or lower gdp or lower gnp or lower gross domestic or lower gross national or lmic or lmics or third world or lami countr* or transitional countr* or emerging economies or emerging nation (Topic)

Scopus Search

TITLE-ABS-KEY (afghanistan OR albania OR algeria OR "American samoa" OR angola OR "antigua and barbuda" OR antigua OR barbuda OR argentina OR armenia OR armenian OR aruba OR azer aijan OR bahrain OR bangladesh OR barbados OR "republic of belarus" OR belarus OR byelarus OR belorussia OR byelorussian OR belize OR "british honduras" OR benin OR dahomey OR bhutan OR bolivia OR "bosnia and herzegovina" OR bosnia OR herzegovina OR botswana OR bechuanaland OR brazil OR brasil OR bulgaria OR "burkina faso" OR "burkina fasso" OR "upper volta" OR burundi OR urundi OR "cabo verde" OR "cape

verde" OR cambodia OR kampuchea OR "khmer
republic" OR cameroon OR cameron OR cameroun OR "central african
republic" OR "ubangi
shari" OR chad OR chile OR china OR colombia OR comoros OR "comoro
islands" OR "iles comores" OR mayotte OR "democratic republic of the
congo" OR "democratic republic congo" OR congo OR zaire OR "costa rica" OR "cote
d'ivoire" OR "cote d' ivoire" OR "cote divoire" OR "cote d ivoire" OR "ivory
coast" OR croatia OR cuba OR cyprus OR "czech
republic" OR czechoslovakia OR djibouti OR "french
somaliland" OR dominica OR "dominican republic" OR ecuador OR egypt OR "united
arab republic" OR "el salvador" OR "equatorial guinea" OR "Spanish
guinea" OR eritrea OR estonia OR eswatini OR swaziland OR ethiopia OR fiji OR g
abon OR "gabonese republic" OR gambia OR "georgia
(republic)" OR georgia OR georgian OR ghana OR "gold
coast" OR gibraltar OR greece OR grenada OR guam OR guatemala OR guinea OR
"guinea bissau" OR guyana OR "british
guiana" OR haiti OR hispaniola OR honduras OR hungary OR india OR indonesia O
R timor OR iran OR iraq OR "isle of
man" OR jamaica OR jordan OR kazakhstan OR kazakh OR kenya OR "democratic
people's republic of korea" OR "republic of
korea" OR north AND korea OR south AND korea OR korea OR kosovo OR kyrgyzst
an OR kirghizia OR kirgizstan OR "kyrgyz republic" OR kirghiz OR laos OR "lao
pdr" OR "lao people's democratic republic" OR latvia OR lebanon OR "lebanese
republic" OR lesotho OR basutoland OR liberia OR libya OR "libyan arab
jamahiriya" OR lithuania OR macau OR macao OR "republic of north
macedonia" OR macedonia OR madagascar OR "malagasy
republic" OR malawi OR nyasaland OR malaysia OR "malay federation" OR "malaya
federation" OR maldives OR "indian ocean islands" OR "indian
ocean" OR mali OR malta OR micronesia OR "federated states of
micronesia" OR kiribati OR "marshall islands" OR nauru OR "northern mariana
islands" OR palau OR tuvalu OR mauritania OR mauritius OR mexico OR moldova
OR moldovian OR mongolia OR montenegro OR morocco OR ifni OR mozambique
OR "portuguese east
africa" OR myanmar OR burma OR namibia OR nepal OR "netherlands
antilles" OR nicaragua OR niger OR nigeria OR oman OR muscat OR pakistan OR
panama OR "papua new
guinea" OR paraguay OR peru OR philippines OR philipines OR phillipines OR philli
ppines OR poland OR "polish people's republic" OR portugal OR "portuguese
republic" OR "puerto rico" OR romania OR russia OR "russian
federation" OR ussr OR "soviet union" OR "union of soviet socialist
republics" OR rwanda OR ruanda OR samoa OR "pacific
islands" OR polynesia OR "samoan islands" OR "navigator island" OR "navigator
islands" OR "sao tome and principe" OR "saudi
arabia" OR senegal OR serbia OR seychelles OR "sierra
leone" OR slovakia OR "slovak republic" OR slovenia OR melanesia OR "solomon
island" OR "solomon islands" OR "norfolk island" OR "norfolk
islands" OR somalia OR "south africa" OR "south sudan" OR "sri
lanka" OR ceylon OR "saint kitts and nevis" OR "st. kitts and nevis" OR "saint
lucia" OR "st. lucia" OR "saint vincent and the grenadines" OR "saint vincent" OR "st.
vincent" OR grenadines OR sudan OR suriname OR surinam OR "dutch
guiana" OR "netherlands guiana" OR syria OR "syrian arab

republic" OR tajikistan OR tadjikistan OR tadzhikistan OR tadzhik OR tanzania OR tanganyika OR thailand OR siam OR "timor leste" OR "east timor" OR togo OR "togolese republic" OR tonga OR "trinidad and tobago" OR trinidad OR tobago OR tunisia OR turkey OR turkmenistan OR turkmen OR uganda OR ukraine OR uruguay OR uzbekistan OR uzbek OR vanuatu OR "new hebrides" OR venezuela OR vietnam OR "viet nam" OR "middle east" OR "west bank" OR gaza OR palestine OR yemen OR yugoslavia OR zambia OR zimbabwe OR "northern rhodesia" OR "global south" OR "africa south of the sahara" OR "sub saharan africa" OR "subsaharan africa" OR "africa, central" OR "central africa" OR "africa, northern" OR "north africa" OR "northern africa" OR magreb OR maghrib OR sahara OR "africa, southern" OR "southern africa" OR "africa, eastern" OR "east africa" OR "eastern africa" OR "africa, western" OR "west africa" OR "western africa" OR "west indies" OR "indian ocean islands" OR caribbean OR "central america" OR "latin america" OR "south and central america" OR "south america" OR "asia, central" OR "central asia" OR "asia, northern" OR "north asia" OR "northern asia" OR "asia, southeastern" OR "southeastern asia" OR "south eastern asia" OR "southeast asia" OR "south east asia" OR "asia, western" OR "western asia" OR "europe, eastern" OR "east europe" OR "eastern europe" OR "developing country" OR "developing countries" OR "developing nation" OR "developing nations" OR "developing population" OR "developing populations" OR "developing world" OR "less developed country" OR "less developed countries" OR "less developed nation" OR "less developed nations" OR "less developed population" OR "less developed populations" OR "less developed world" OR "lesser developed country" OR "lesser developed countries" OR "lesser developed nation" OR "lesser developed nations" OR "lesser developed population" OR "lesser developed populations" OR "lesser developed world" OR "under developed country" OR "under developed countries" OR "under developed nation" OR "under developed nations" OR "under developed population" OR "under developed populations" OR "under developed world" OR "underdeveloped country" OR "underdeveloped countries" OR "underdeveloped nation" OR "underdeveloped nations" OR "underdeveloped population" OR "underdeveloped populations" OR "underdeveloped world" OR "middle income country" OR "middle income countries" OR "middle income nation" OR "middle income nations" OR "middle income population" OR "middle income populations" OR "low income country" OR "low income countries" OR "low income nation" OR "low income nations" OR "low income population" OR "low income populations" OR "lower income country" OR "lower income countries" OR "lower income nation" OR "lower income nations" OR "lower income population" OR "lower income populations" OR "underserved country" OR "underserved countries" OR "underserved nation" OR "underserved nations" OR "underserved population" OR "underserved populations" OR "underserved world" OR "under served country" OR "under served countries" OR "under served nation" OR "under served nations" OR "under served population" OR "under served populations" OR "under served world" OR "deprived country" OR "deprived countries" OR "deprived nation" OR "deprived nations" OR "deprived population" OR "deprived populations" OR "deprived world" OR "poor country" OR "poor countries" OR "poor nation" OR "poor nations" OR "poor population" OR "poor populations" OR "poor world" OR "poorer country" OR "poorer countries" OR "poorer nation" OR "poorer nations" OR "poorer population" OR "poorer populations" OR "poorer world" OR "developing economy" OR "developing economies" OR "less developed economy" OR "less developed economies" OR "lesser developed economy" OR "lesser

developed economies" OR "under developed economy" OR "under developed economies" OR "underdeveloped economy" OR "underdeveloped economies" OR "middle income economy" OR "middle income economies" OR "low income economy" OR "low income economies" OR "lower income economy" OR "lower income economies" OR "low gdp" OR "low gnp" OR "low gross domestic" OR "low gross national" OR "lower gdp" OR "lower gnp" OR "lower gross domestic" OR "lower gross national" OR lmic OR lmics OR "third world" OR "lami country" OR "lami countries" OR "transitional country" OR "transitional countries" OR "emerging economies" OR "emerging nation" OR "emerging nations") AND ((TITLE-ABS-KEY (dementia OR alzheimer* OR "Lewy bod*" OR "Frontotemporal" OR "Dement*" OR "Vascular dementia" OR "cognitive* degeneration" OR "cognitive impairment") AND TITLE-ABS-KEY (carer* OR caregive*) AND TITLE-ABS-KEY (informal OR unpaid OR famil*) AND TITLE-ABS KEY (support OR training OR intervention OR course OR trial OR education OR awareness)))

Google Scholar

dementia education carers LMICs

dementia education training carers caregivers LMICs

dementia Alzheimer's intervention carers caregivers LMICs

Table S1 - Overview of Quality Assessment

**Item has been modified- score as Yes/No/Unable to determine.*

	Baruah et al., 2021	Dias et al., 2008	Fialho et al., 2012	Gavrilova et al., 2009	Guerra et al., 2011	Han et al., 2022	Hinton et al., 2020	Javadpour et al., 2009	Kuzu et al., 2005	Magteppong and Yamarat, 2021	Pankong et al., 2018	Santos et al., 2013	Senanarong et al., 2004	Shata et al., 2017	Tawfik et al., 2021	Tran et al., 2022	Zakaria and Ab Razak, 2017	Zhang et al., 2020
1. Clear hypotheses/aims/objectives?	Y	Y	Y	Y	Y	Y	Y	Y	Y	Y	Y	Y	Y	Y	Y	Y	Y	Y
2. Outcome to be measures in the introduction/method?	Y	Y	Y	Y	Y	Y	Y	Y	Y	Y	Y	Y	Y	Y	Y	Y	Y	Y
3. Participant characteristics described? (inclusion/exclusion)	Y	Y	Y	Y	Y	Y	Y	N	N	Y	Y	N	Y	Y	Y	Y	Y	Y
4. Interventions of interest clearly described?	Y	Y	Y	Y	Y	Y	Y	Y	Y	Y	Y	Y	Y	Y	Y	Y	Y	Y
5. Principle cofounders described?	Y	Y	Y	Y	Y	Y	Y	Y	Y	Y	Y	Y	Y	Y	Y	Y	Y	Y
6. Main findings clearly described?	Y	Y	Y	Y	Y	Y	Y	Y	Y	Y	Y	Y	Y	Y	Y	Y	Y	Y
7. Estimates of the random variability of the data for the main outcomes provided?	Y	Y	Y	Y	Y	Y	Y	Y	Y	Y	Y	Y	Y	Y	Y	Y	Y	Y

8. All important adverse events that may be a consequence of intervention reported?	N	N	N	N	N	N	N	N	N	N	N	N	N	N	N	N	N	N
9. Characteristics of participants lost to follow-up described?	Y	Y	Y	Y	Y	N	Y	Y	Y	Y	Y	Y	Y	Y	Y	Y	Y	Y
10. Actual probability values reported for main outcomes? (Except if <.001)	Y	N	Y	Y	Y	Y	Y	Y	Y	Y	Y	Y	Y	Y	Y	Y	Y	Y
11. Subjects asked to participate representative of entire population?	Y	Y	U	Y	Y	Y	Y	U	Y	Y	Y	U	Y	Y	Y	Y	Y	Y
12. Subjects prepared to participate representative of entire population?	Y	Y	Y	Y	Y	Y	Y	N	Y	Y	Y	Y	Y	Y	Y	Y	Y	Y
13. Staff, places, and facilities where the patients were treated, representative of the treatment the majority of patients receive?	N	Y	Y	Y	Y	N	Y	Y	Y	Y	U	U	Y	N	Y	Y	Y	Y
14. Attempt made to blind study subjects to intervention received?	Y	Y	N	Y	Y	N	N	N	N	U	U	N	U	Y	U	N	N	N
15. Attempt made to blind those measuring the main outcomes?	U	Y	N	Y	Y	N	Y	N	N	U	U	N	U	Y	U	Y	N	U
16. Results based on "data dredging" made clear?	Y	Y	Y	Y	Y	Y	Y	U	Y	Y	Y	Y	Y	Y	Y	Y	Y	Y
17. In trials and cohort studies, analyses adjust for different lengths of follow-up, or in case-control studies, time between	Y	Y	Y	Y	Y	Y	Y	Y	Y	Y	Y	Y	Y	Y	Y	Y	Y	Y

the intervention and outcome the same for cases and controls ?																		
18. Appropriate statistical tests?	Y	Y	Y	Y	Y	Y	Y	Y	Y	Y	Y	Y	Y	Y	Y	Y	Y	Y
19. Compliance with the intervention/s reliable?	N	U	Y	U	U	U	Y	U	U	U	Y	Y	Y	U	U	Y	Y	U
20. Main outcomes accurate (valid and reliable)?	Y	Y	Y	Y	Y	Y	Y	Y	Y	Y	Y	Y	Y	Y	Y	U	Y	Y
21. Participants in different conditions recruited from same population?	Y	Y	U	Y	Y	Y	Y	U	Y	Y	Y	Y	Y	Y	Y	Y	Y	Y
22. Participants in different conditions recruited over same time period?	Y	Y	U	Y	Y	Y	Y	U	Y	Y	Y	Y	Y	Y	Y	Y	Y	Y
23. Participants randomised?	Y	Y	N	Y	Y	N	Y	N	N	Y	Y	N	Y	Y	Y	Y	N	N
24. Randomisation concealed from both participants and staff until recruitment was complete?	U	Y	N	Y	Y	N	Y	N	N	U	U	N	U	Y	Y	N	N	N
25. Adequate adjustment for confounding in the analyses?	Y	Y	N	Y	Y	Y	Y	N	N	Y	Y	N	Y	Y	Y	Y	N	Y
26. Losses of participants to follow-up taken into account?	N	Y	Y	Y	Y	U	Y	Y	Y	Y	Y	Y	Y	Y	Y	Y	N	Y
27. Sufficient power to detect an effect? (<5% likelihood due to chance)*	N	N	N	Y	Y	N	U	N	N	Y	Y	U	U	Y	Y	U	N	U
**Score**	**20**	**23**	**17**	**25**	**25**	**17**	**24**	**13**	**18**	**22**	**22**	**17**	**22**	**24**	**23**	**22**	**19**	**20**
